# Deep learning model for analyzing the relationship between mandibular third molar and inferior alveolar nerve in panoramic radiography

**DOI:** 10.1038/s41598-022-21408-9

**Published:** 2022-10-08

**Authors:** Shintaro Sukegawa, Futa Tanaka, Takeshi Hara, Kazumasa Yoshii, Katsusuke Yamashita, Keisuke Nakano, Kiyofumi Takabatake, Hotaka Kawai, Hitoshi Nagatsuka, Yoshihiko Furuki

**Affiliations:** 1 Department of Oral and Maxillofacial Surgery, Kagawa Prefectural Central Hospital, 1-2-1, Asahi-machi, Takamatsu, Kagawa 760-8557 Japan; 2 Department of Oral and Maxillofacial Surgery, Kagawa University School of Medicine, 1750-1 Ikenobe, Miki, Kagawa 761-0793 Japan; 3 Department of Oral Pathology and Medicine, Graduate School of Medicine, Dentistry and Pharmaceutical Sciences, Okayama University, Okayama, 700-8558 Japan; 4 Department of Electrical, Electronic and Computer Engineering, Faculty of Engineering, Gifu University, 1-1 Yanagido, Gifu, Gifu 501-1193 Japan; 5 Center for Healthcare Information Technology (C-HiT), Tokai National Higher Education and Research System, 1-1 Yanagido, Gifu, Gifu 501-1193 Japan; 6 Polytechnic Center Kagawa, 2-4-3, Hananomiya-cho, Takamatsu, Kagawa 761-8063 Japan

**Keywords:** Dentoalveolar surgery, Digital radiography in dentistry, Panoramic radiography

## Abstract

In this study, the accuracy of the positional relationship of the contact between the inferior alveolar canal and mandibular third molar was evaluated using deep learning. In contact analysis, we investigated the diagnostic performance of the presence or absence of contact between the mandibular third molar and inferior alveolar canal. We also evaluated the diagnostic performance of bone continuity diagnosed based on computed tomography as a continuity analysis. A dataset of 1279 images of mandibular third molars from digital radiographs taken at the Department of Oral and Maxillofacial Surgery at a general hospital (2014–2021) was used for the validation. The deep learning models were ResNet50 and ResNet50v2, with stochastic gradient descent and sharpness-aware minimization (SAM) as optimizers. The performance metrics were accuracy, precision, recall, specificity, F1 score, and area under the receiver operating characteristic curve (AUC). The results indicated that ResNet50v2 using SAM performed excellently in the contact and continuity analyses. The accuracy and AUC were 0.860 and 0.890 for the contact analyses and 0.766 and 0.843 for the continuity analyses. In the contact analysis, SAM and the deep learning model performed effectively. However, in the continuity analysis, none of the deep learning models demonstrated significant classification performance.

## Introduction

Third molar extraction is the most common surgery performed by dentists and maxillofacial surgeons. The mandibular third molar has more complications than the maxillary third molar, including post-extraction infection, postoperative pain, and inferior alveolar nerve damage^1,2^. Among these complications, inferior alveolar nerve damage should be avoided because it stresses patients for a prolonged period.

In clinical practice, panoramic radiographs are generally used to determine the difficulty of the mandibular third molar, including the contact with the inferior alveolar nerve, depth of the mandibular third molar, and distance to the mandibular ramus. If contact between the inferior alveolar canal and mandibular third molar is suspected after the panoramic screening, computed tomography (CT) is used to identify defects in the cortical bone around the inferior alveolar nerve. The cortical bone defects are significantly risky for postoperative inferior alveolar nerve damage^3^. Accurately determining the positional relationship between the inferior alveolar nerve and the mandibular third molar with a two-dimensional panoramic radiograph is difficult, but preoperative diagnosis using CT in 3D is very effective^4^. Although CT cannot directly image the nerve, it can clarify the positional relationship between the tooth and the nerve by depicting the inferior alveolar nerve and the bony border^5^. However, CT imaging cannot be applied in all cases owing to radiation exposure and high cost^6^. Therefore, developing an assistant diagnostic tool to diagnose the contact relationship with the inferior alveolar nerve from panoramic radiograph images is essential.

Convolutional neural networks (CNN) have revolutionized deep learning in recent years. CNN-based classifiers have proved highly accurate for image recognition^7^ and have consequently impacted diagnostic imaging in the medical field. They have been applied to the detection of lung cancer from chest X-ray images^8^, determination of retinal detachment^9^, detection of osteoporosis^10^, screening of breast cancer^11^, etc. In addition, many deep learning-related studies have been reported in the field of dentistry, and classifiers have been developed for areas such as caries^12^, periapical lesions^13^, dental implants^14^, maxillary sinusitis^15^, and position classification of the mandibular third molars^16^. Furthermore, deep learning has occasionally been more accurate than human diagnosis^17,18^. In contact analysis using deep learning, Fukuda et al.^19^ examined images of different sizes and reported that the results were more accurate when the images were small and condensed only to those that required a large amount of information. Thus, deep learning diagnostic imaging has great potential and must be explored. We therefore hypothesized that deep learning could accurately diagnose the positional relationship between the mandibular third molar and inferior alveolar nerve on panoramic radiographs.

This study aimed to explain the accuracy of the positional relationship of the contact between the inferior alveolar canal and the mandibular third molar using deep learning. To this end, in this CNN deep learning-based study, we first investigated the diagnostic performance of the presence or absence of contact between the mandibular third molar and inferior alveolar canal. Subsequently, we explored the diagnostic performance of bone continuity between the mandibular third molar and inferior alveolar canal, diagnosed using CT.

## Materials and methods

### Study design

This study analyzed the diagnostic performance of the positional relationship between the inferior alveolar canal/nerve and the mandibular third molar from panoramic radiographs using an optimized CNN deep learning model.

### Ethics statement

This study was approved by the Institutional Review Board of Kagawa Prefectural Central Hospital (approval number: 1023; approval date: 8th March 2021). The board reviewed our retrospective non-interventional study design and analytical study with anonymized data and waived written documentation of personal informed consent. All methods were performed following the relevant guidelines and regulations. The study was registered at jRCT (jRCT1060220021).

### Preparation of image datasets

We retrospectively used radiographic imaging data collected at the Department of Oral and Maxillofacial Surgery in a single general hospital from April 2014 to December 2021. The study data included patients aged 20–76 years in the mature mandibular third molar who had panoramic radiographs and CT taken on the same day. This study confirms the positional relationship between the mandibular third molar and the inferior alveolar canals by panoramic radiography. An unclear image (three teeth) and an image of the remaining titanium plate after the mandibular fracture (one tooth) were excluded. Finally, 1279 tooth images were used in this study.

**Figure 1:**
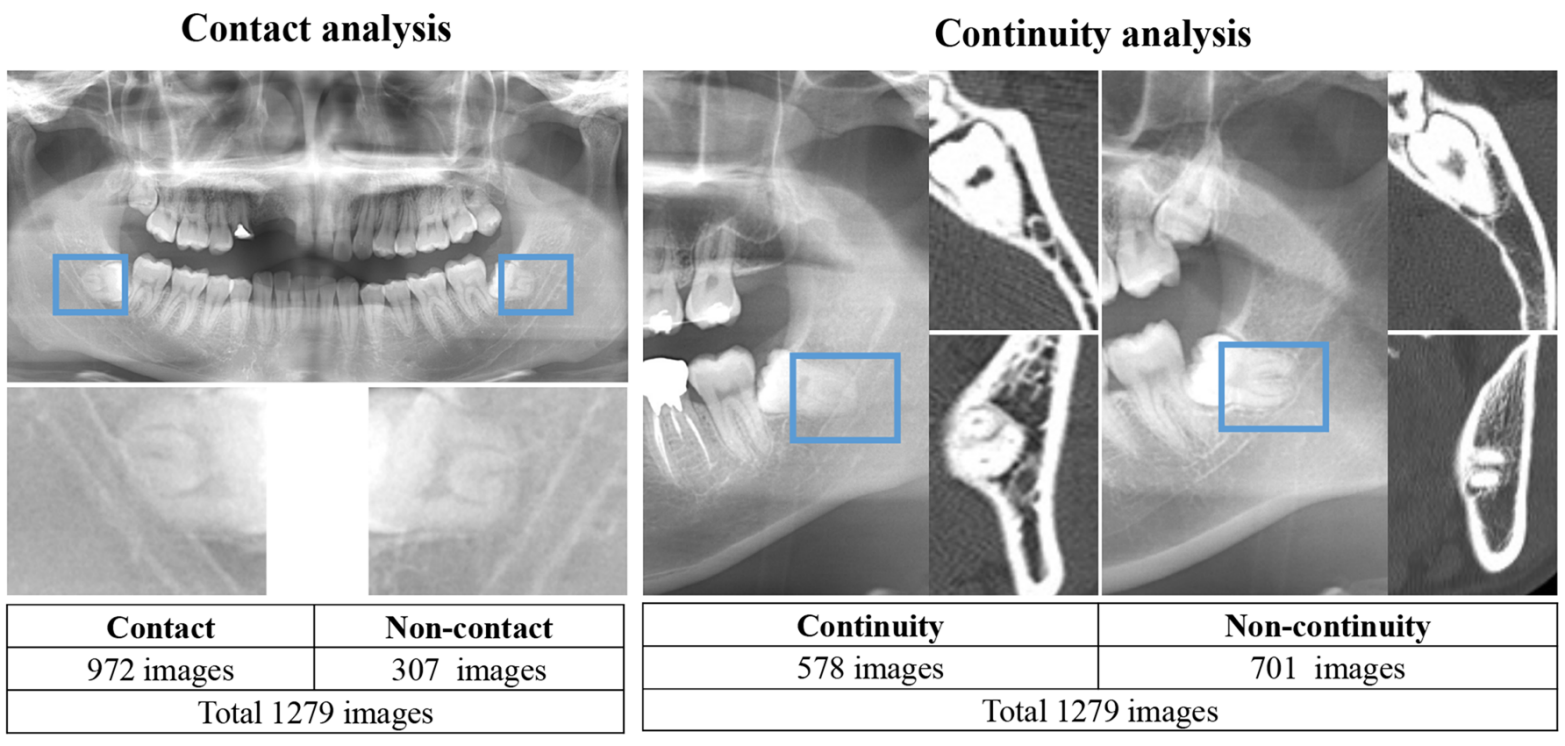
Classification of *the* relationship *between* the mandibular third molar and inferior alveolar canal/nerve.

Digital image data were obtained using dental panoramic radiographs taken with either of the two imaging devices (AZ3000CMR or Hyper-G CMF; ASAHIRENTOGEN Ind. Co., Ltd., Kyoto, Japan). All digital image data were output in a tagged image file format (digital image size: 2776 × 1450, 2804 × 1450, 2694 × 1450, or 2964 × 1464 pixels) using the Kagawa Prefectural Central Hospital Picture Archiving and Communication Systems system (Hope Dr. Able-GX, Fujitsu Co., Tokyo, Japan). Under the supervision of an expert oral and maxillofacial surgeon, two oral and maxillofacial surgeons used Photoshop Elements (Adobe Systems, Inc., San Jose, CA, USA) to crop the areas of interest manually. The image was cropped by selecting the area, including the apex of the mandibular third molar and the inferior alveolar canal within 250 × 200 pixels (Fig. 1). Each cropped image had a resolution of 96 dpi and was saved in the portable network graphic format.

### Classification of mandibular third molar and inferior alveolar nerve

First, using panoramic radiographs, we classified the contact and superimposition between the mandibular third molar and inferior alveolar canal. This is because contact between the inferior alveolar duct on panoramic radiographs is a risk factor for nerve exposure^4,20^.

Second, we classified the presence or absence of direct contact between the mandibular third molar and inferior alveolar nerve using CT. The classification criteria and distributions are as follows and are also shown in Fig. 1.

1. Relationship between the mandibular third molar and inferior alveolar canal.
  - Non-contact or superimposition of the mandibular third molar and inferior alveolar canal.
  - Contact or superimposition between the mandibular third molar and the inferior alveolar canal.

In this study, contacts and superimposition/overlaps were grouped together.

2. Relationship between the mandibular third molar and inferior alveolar nerve.

If there was discontinuity of the cortical bone at the inferior alveolar canal due to the mandibular third molar, it was classified as a defect.

- Contact between the mandibular third molar and inferior alveolar nerve (i.e., defect or discontinuity in the cortical bone of the inferior alveolar canal).
- Non-contact between the mandibular third molar and inferior alveolar nerve (i.e., continuity of the cortical bone of the inferior alveolar canal).

### CNN model architecture

**Figure 2:**
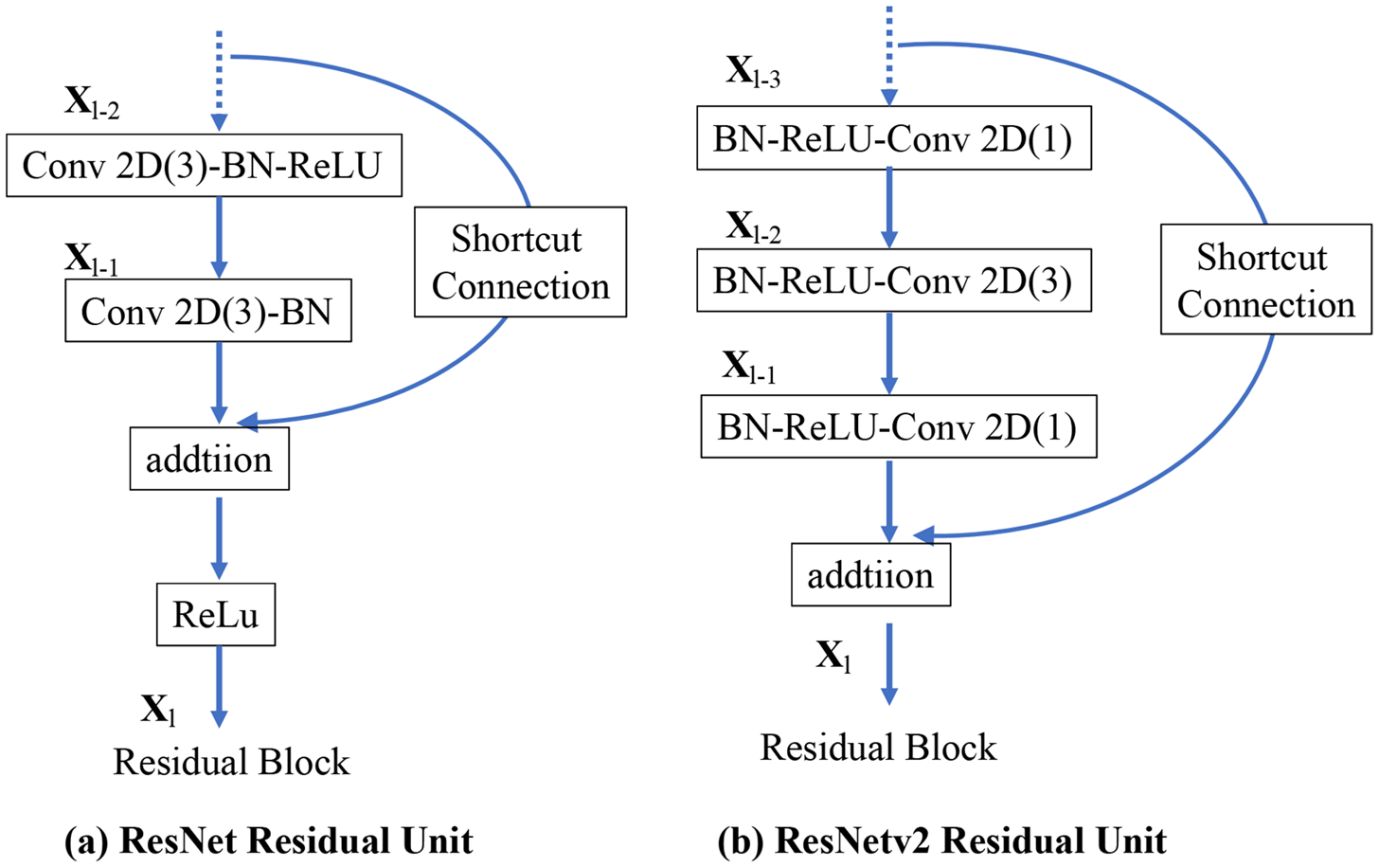
Differences between the residual blocks of ResNet and ResNetv2: (**a**) ResNet Residual Unit; (**b**) ResNetv2 Residual Unit. BN: Batch Normalization and Conv2D: Two-dimensional convolution layer.

ResNet50 is a 50-layer deep CNN model. Traditional CNNs have the major drawback of the “vanishing gradient problem,” where the gradient value is significantly reduced during backpropagation, resulting in little weight change. The ResNet CNN model uses a residual module to overcome this problem^21^. ResNet v2 is an improved version of the original ResNet^22^, with the following improvements compared with the original ResNet (Fig. 2): (1) The shortcut path is completely identity mapped without using the ReLU between the input and output. (2) After branching for the residual calculation, the order is changed to batch normalization^23^ as -ReLU-convolution-batch normalization-convolution.

In this study, we selected two CNN models, ResNet50 and ResNet50v2. The ResNet50 and ResNet50v2 CNN models were pre-trained on the ImageNet database and fine-tuned according to the positional relationship classification task for the mandibular third molar and inferior alveolar nerve. The deep learning task process was implemented using Python (version 3.7.13), Keras (version 2.8.0), and TensorFlow (version 2.8.0).

### Dataset and CNN model training

Each CNN model training was generalized using K-fold cross-validation in the deep learning algorithm. The models were validated using tenfold cross-validation to ensure internal validity. The digital image dataset was divided into ten random subsets using the stratified sampling technique, and the same classification distribution was maintained for training, validation, and testing across all subsets^24^. The dataset was split into separate test and training datasets in a ratio of 0.1–0.9 within each fold. Additionally, the validation data comprised one-tenth of the training dataset. The model averaged ten training iterations to obtain prediction results for the entire dataset, with each iteration retaining a different subset for validation.

The cross-entropy—defined by Eq. (1)—was used for the loss function.

1 $$L= -\sum\limits_{i=0}^{n}{t}_{i}\mathrm{log}{y}_{i}$$

where t_*i*_ is true label and y_*i*_ is the predicted probability of class *i*.

#### Optimization algorithm

This study used two deep learning gradient methods, stochastic gradient descent (SGD) and sharpness-aware minimization (SAM). SGD is a typical optimization method in which the parameters are updated by the magnitude in the obtained gradient direction. The momentum SDG is a method of adding momentum to SGD^25^. In this study, the momentum was set to 0.9. The momentum SGD is expressed in Eqs. (2) and (3).

2 $$\Delta {w}_{t}=\eta \nabla \mathrm{L}\left(\mathrm{w}\right)+\mathrm{\alpha }\Delta {w}_{t-1}$$

3 $${w}_{t}={w}_{t-1}-\Delta {w}_{t}$$

where w_t is t-th parameter, η is learning rate, ∇L (w) is differentiation with parameters of the loss function, and α is momentum.

SAM is an optimization method that converges to a parameter with minimal loss and flat surroundings^26^. It uses a combination of a base optimizer and SAM to determine the final parameters using traditional algorithms. SGD was selected as the base optimizer. The loss function of SAM is defined by Eq. (4). This is used to minimize Eq. (5).

4 $$\underset{w}{\mathrm{min}}{L}_{S}^{SAM}\left(w\right)+\lambda {\Vert w\Vert }_{2}^{2}$$

5 $${L}_{S}^{SAM}\left(w\right)=\underset{{\Vert \varepsilon \Vert }_{p}\le \rho }{\mathrm{max}}{L}_{s}(w+\varepsilon )$$

where S is the set of data, w is a parameter, λ is the L2 regularization coefficient, L_s is the loss function, and ρ is the neighborhood size.

This study analyzed the deep learning models using a ρ value of 0.025.

### Deep learning procedure

#### Data augmentation

Data augmentation prevents excessive adaptation to the training data by diversifying the input data^27^. The following values were selected for the preprocessing layer to convert the images during training randomly. The boundary surface of the missing part was complemented by folding back using the reflect method.

- Random rotation: range of − 18° to 18°
- Random flip: horizontally and vertically
- Random translation: up–down and left–right range of 30%

#### Learning rate scheduler

Learning rate decay is a technique used to improve the generalization performance of deep learning and reduce the learning rate from a state in which learning has progressed to some extent. Decay in the learning rate can improve accuracy^21^. The changes due to time-based decay as a learning rate can be found in the appendix. The learning rate decay can be evaluated using Eq. (6).

6 $${ lr}_{new}= \frac{{lr}_{current}}{(1+decay\ rate\times epoch)}$$

The learning rate scheduler was executed with an initial learning rate of 0.01 and a decay rate of 0.001. All the models conducted analysis over 300 epochs and with 32 batch sizes without early stopping. These deep learning processes were repeated 30 times for all models using different random seeds for each analysis.

### Performance metrics and statistical analysis

To evaluate the performance of each deep learning model, the accuracy, precision, recall, F1 score, and area under the curve (AUC)—calculated from the receiver operating characteristic (ROC) curve—performance metrics were employed. More detailed information on the performance metrics used in this study is present in the Appendix.

Statistical evaluations of the performance for each deep learning model were performed on the data that were independently and repeatedly analyzed 30 times. Data were recorded and stored in an electronic database using Microsoft Excel (Microsoft Inc., Redmond, WA, USA). The database was created and analyzed by using JMP Statistical Software Package Version 14.2.0 for Macintosh (SAS Institute Inc., Cary, NC, USA). All statistical analyses were bilateral with a significance level of 0.05. Normal distribution was evaluated by using the Shapiro–Wilk test. A comparison of classification performance between each CNN model was performed for each metric by using the Wilcoxon signed rank sum test. Effect sizes^28^ were evaluated using Hedges' g (unbiased Cohen's d), Eqs. (7) and (8).

7 $$ Hedges^{\prime}g = \frac{{|M_{1} - M_{2} |}}{s} $$

8 $$s=\sqrt{\frac{{(n}_{1}-1){s}_{1}^{2}+({n}_{2}-1){s}_{2}^{2}}{{n}_{1}+{n}_{2}-2}}$$

where M1 and M2 are the mean values for the CNN models with SGD and SAM, s1 and s2 are the standard deviations for the CNN models with SGD and SAM, respectively; and n1 and n2 are the numbers for the CNN models with SGD and SAM, respectively. Effect sizes were categorized as large effect, ≥ 2.0; very large effect, 1.0; large effect, 0.8; medium effect, 0.5; small effect, 0.2; and very small effect, 0.01 based on the criteria proposed by Cohen and extended by Sawilowsky^29^.

### Visualization of judgment regions in deep learning

In this study, the gradient-weighted class activation map (Grad-CAM) algorithm^30^ was used to visualize the noticeable areas of the image in a heatmap. Grad-CAM is a class activation mapping method that uses gradients for weights adopted by the IEEE International Conference on Computer Vision in 2017 and provides a visual basis for deep learning to improve the explanation of the architecture. Grad-CAM uses the last convolution layer of the ResNet model to visualize the feature area.

## Results

### Performance metrics of ResNet50 and ResNet50v2 in the SAM and SGD optimizers

Table 1 shows the results of the performance metrics of ResNet50 and ResNet50v2 with the SAM and SGD optimizers in the contact analysis. In the contact analysis of the inferior alveolar canal and mandibular third molar on panoramic radiographic images, ResNet 50v2 using the SAM optimizer showed the highest performance on all performance metrics (Accuracy: 0.860, Precision: 0.816, Recall: 0.791, F1 score: 0.800, and AUC: 0.890).

**Table 1:** Performance metrics of ResNet50 and ResNet50v2 with the SAM and SGD optimizers in contact analysis.

CNN	Optimizer	Accuracy	Precision	Recall	F1 score	AUC
		SD	SD	SD	SD	SD
		95%CI	95% CI	95% CI	95% CI	95% CI
ResNet50	SAM	0.855	0.810	0.785	0.794	0.883
		0.005	0.009	0.009	0.008	0.007
		0.853–0.857	0.807–0.813	0.782–0.789	0.791–0.797	0.880–0.885
ResNet50	SGD	0.850	0.804	0.781	0.789	0.875
		0.009	0.010	0.010	0.009	0.008
		0.847–0.853	0.800–0.807	0.785–0.778	0.786–0.793	0.872–0.878
ResNet50v2	SAM	0.860	0.816	0.791	0.800	0.890
		0.005	0.008	0.009	0.008	0.007
		0.858–0.861	0.813–0.819	0.788–0.794	0.798–0.803	0.888–0.893
ResNet50v2	SGD	0.853	0.809	0.782	0.792	0.884
		0.005	0.008	0.009	0.007	0.006
		0.851–0.855	0.806–0.812	0.779–0.785	0.790–0.795	0.882–0.886

SD, standard deviation; 95% CI, 95% confidence interval; AUC, area under the receiver operating characteristics curve.

Table 2 shows the results of the performance metrics of ResNet50 and ResNet50v2 with the SAM and SGD optimizers in the continuity analysis. In the continuity analysis of the inferior alveolar nerve and mandibular third molar on panoramic radiographic images, ResNet50v2 using the SAM optimizer showed the highest performance on all performance metrics and contact analysis (Accuracy: 0.766, Precision: 0.766, Recall: 0.765, F1 score: 0.775, and AUC: 0.843).

**Table 2:** Performance metrics of ResNet50 and ResNet50v2 with optimizers SAM and SGD continuity analysis.

CNN	Optimizer	Accuracy	Precision	Recall	F1 score	AUC
		SD	SD	SD	SD	SD
		95% CI	95% CI	95% CI	95% CI	95% CI
ResNet50	SAM	0.754	0.755	0.754	0.753	0.832
		0.005	0.008	0.008	0.008	0.006
		0.753–0.756	0.752–0.757	0.751–0.757	0.750–0.755	0.829–0.834
ResNet50	SGD	0.754	0.754	0.754	0.752	0.830
		0.007	0.008	0.008	0.008	0.006
		0.752–0.757	0.752–0.757	0.751–0.757	0.750–0.755	0.827–0.832
ResNet50v2	SAM	0.766	0.766	0.765	0.775	0.843
		0.007	0.006	0.006	0.013	0.005
		0.764–0.769	0.764–0.768	0.763–0.767	0.771–0.780	0.842–0.845
ResNet50v2	SGD	0.765	0.765	0.765	0.767	0.842
		0.006	0.006	0.006	0.013	0.005
		0.763–0.768	0.763–0.767	0.762–0.767	0.762–0.772	0.840–0.844

SD, standard deviation; 95% CI, 95% confidence interval; AUC, area under the receiver operating characteristics curve.

### Statistical evaluation of performance metrics in each CNN model

Tables 3 and 4 show the statistical evaluation results of both CNN models for each performance metric. Contact and continuity analyses yielded symmetrical results. For the contact analysis results shown in Table 3, both ResNet50 and ResNet50v2 exhibited statistically significant differences on all performance metrics for SAM and SGD. AUC and accuracy for ResNet50 showed the highest effect size equivalent to “very large” using SAM. The comparison of ResNet50v2 and ResNet50 using SAM showed a statistically significantly higher performance for ResNet50v2 on all performance metrics.

**Table 3:** Statistical evaluation of ResNet50 and ResNet50v2 with the SAM and SGD optimizers in contact analysis.

Performance metrics	Model A	Model B	A-B	P value	Effect size
**ResNet50**					
Accuracy	SAM	SGD	0.006	0.003	0.761
Precision			0.006	0.006	0.677
Recall			0.004	0.046	0.440
F1 score			0.005	0.018	0.556
AUC			0.008	< .0001	1.052
**ResNet50v2**					
Accuracy	SAM	SGD	0.007	< .0001	1.456
Precision			0.007	< .0001	0.874
Recall			0.009	< .0001	0.995
F1 score			0.008	< .0001	1.103
AUC			0.006	0.001	0.899
**ResNet50v2 versus ResNet50 optimizer; SAM**					
Accuracy	ResNet50v2	ResNet50	0.004	0.004	0.835
Precision			0.006	0.004	0.742
Recall			0.005	0.013	0.560
F1 score			0.006	0.004	0.712
AUC			0.007	< .0001	0.932

AUC, area under the receiver operating characteristics curve.

**Table 4:** Statistical evaluation of ResNet50 and ResNet50v2 with the SAM and SGD optimizers in continuity analysis.

Performance metrics	Model A	Model B	A-B	P value	Effect size
**ResNet50**					
Accuracy	SAM	SGD	0.0001	0.8442	0.0162
Precision			0.0001	0.8629	0.0109
Recall			0.0001	0.8485	0.0119
F1 score			0.0002	0.8176	0.0195
AUC			0.0022	0.0996	0.3512
**ResNet50v2**					
Accuracy	SAM	SGD	0.0007	0.6328	0.1103
Precision			0.0010	0.5389	0.1576
Recall			0.0001	0.9123	0.0214
F1 score			0.0080	0.0523	0.6064
AUC			0.0014	0.2584	0.2737
**ResNet50 versus ResNet50v2 optimizer; SAM**					
Accuracy	ResNet50v2	ResNet50	0.0116	< .0001	1.9765
Precision			0.0112	< .0001	2.3677
Recall			0.0105	< .0001	2.0622
F1 score			0.0225	< .0001	4.6346
AUC			0.0113	< .0001	2.1598

AUC, area under the receiver operating characteristic curve.

For the continuity analysis shown in Table 4, neither ResNet50 nor ResNet50v2 demonstrated a statistically significant difference on any performance metric when comparing SAM and SGD. The effect size was “small” to “very small” for ResNet50. Conversely, a comparison of ResNet50v2 and ResNet50 using SAM demonstrated a statistically higher performance for ResNet50v2 on all performance metrics, and all effect sizes also showed “very large” to “huge.”

### Comparison of the learning curves of the CNN models

**Figure 3:**
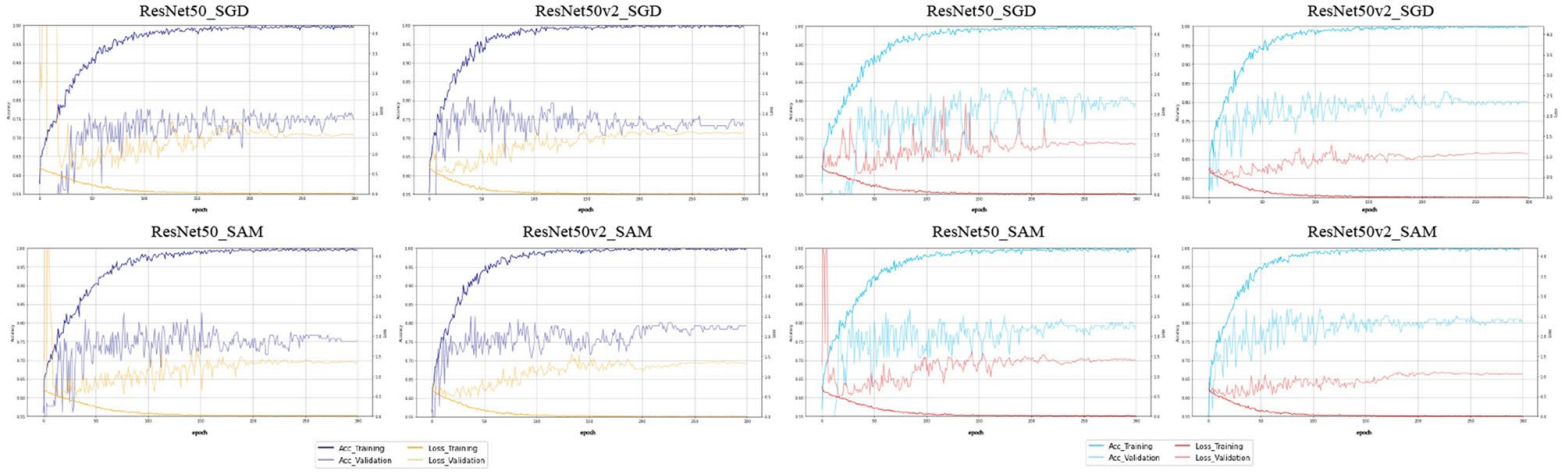
Learning curves for each CNN model in contact and continuity analyses.

Figure 3 shows the learning curve for each CNN deep learning model. In the contact analysis, SGD exhibited a tendency for overfitting with increasing epochs, whereas for the CNN model with SAM, SAM exhibited low overfitting. Interestingly, continuity analysis also demonstrated overfitting for the CNN models using SAM.

### Visualization of model classification by Grad-CAM

**Figure 4:**
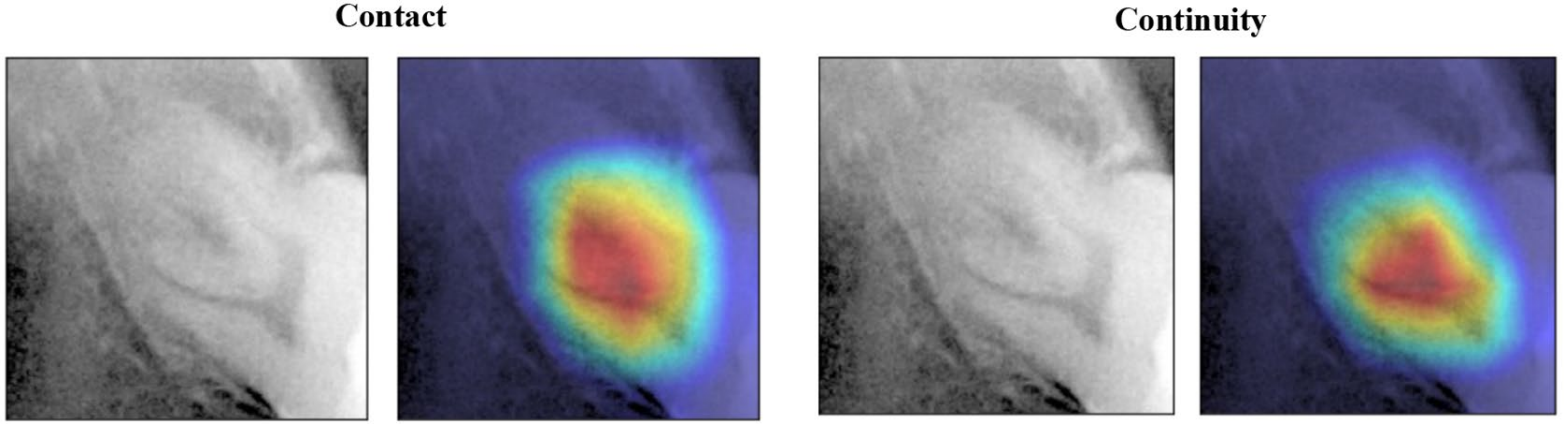
Visualization of regions of interest for CNN classification in contact and continuity analyses.

Figure 4 shows the visualization of the area of interest for classification decisions in each deep learning model in the contact and continuity analyses. In the ResNet50 and ResNet50v2-based CNN models, Grad-CAM visualized the final layer of the convolutional layer or the feature area using a heat map. There was no significant difference in the feature areas indicated by the Grad-CAM in contact and continuity analyses. The point of contact between the inferior alveolar canal and mandibular third molar or the closest part was determined to be the characteristic area. This area of interest was the same as the dentist's judgment. In the heatmap visualization using Grad-CAM, the warmer the color, the more significant the contribution to feature determination.

## Discussion

In this study, we analyzed deep learning models using contact and continuity analyses to classify the positional relationship of the inferior alveolar canal. The CNN model demonstrated a high performance in contact analysis. The CNN model using SAM as the optimizer for ResNet 50v2 exhibited the highest performance. However, in the continuity analysis, none of the CNN models showed high classification performance. This study focused on the part in contact with the inferior alveolar canal and mandibular third molar and obtained a high classification performance using CNN models. However, some cases have been misclassified due to the tooth-like sclerotic area between the teeth and the inferior alveolar canal, and the cropped images made it difficult to identify the inferior alveolar canal. Therefore, optimized data collection is required for contact analysis.

In general, as CT and MRI can provide three-dimensional (3D) information, it is possible to accurately determine the positional relationship between the lower alveolar canal and the mandibular third molar by comparing it with a panoramic image^31^. Analysis using deep learning was performed to determine the 3D positional relationships from panoramic images without using other imaging devices. In continuity analysis, although it is difficult to simply compare performance in deep learning studies conducted on different data, a deep learning classification study conducted on 571 images by Choi et al. reported a classification accuracy of 0.723^32^. The accuracy of the deep learning model in this study was 0.766, which is almost the same as the classification accuracy. In addition, the diagnostic accuracy of specialists was 0.55–0.72 (average 0.63), and it was difficult for even specialists to evaluate the continuity between the inferior alveolar nerve and the mandibular third molar using only panoramic radiographic images. The diagnostic accuracy of deep learning is also equivalent to the highest value among specialists, suggesting CNN models cannot improve the breakthrough diagnosis of continuity.

SGD identifies a point that minimizes the loss function. Although the loss function becomes small, the peripheral optimization parameters become nonuniform. This leads to overfitting and reduced generalization performance. In contrast, in SAM, the loss function is designed to search for flat parameters. Therefore, the values around the selected parameters also exhibited a uniformly low loss function. It improves the generalization performance and robustness against noise. In this study, SGD showed a tendency for overfitting in the contact analysis in comparison to SAM. By contrast, the continuity analysis showed a trend to overfit even in the CNN model using SAM. This is probably due to inconsistencies between the image data and the correct label. In other words, it suggests that even with the deep learning method, it was not possible to identify the absolute feature showing continuity of the inferior alveolar canals. This is the first study to analyze the relationship between the inferior alveolar nerve and mandibular third molars using SAM. The findings of this study will contribute to the development of deep learning in dentistry in the future.

The characteristics of ResNet, a derivative of ResNet50, are (1) input batch normalization and ReLU activation before the convolution operation and (2) nonlinearity creation as an identity mapping. In other words, the output of the additive operation between the identity mapping and residual map can be passed directly to the next block for further processing to facilitate the propagation of information. In this study, the learning curve of ResNet50v2 exhibited a more stable learning process than that of ResNet50. In addition, ResNet50v2 showed a statistically significant improvement in performance metrics in both the contact and continuity analyses, demonstrating that it is an optimal CNN model.

One problem with deep learning is that the inference process for the input data is a black box and the reason for extracting the features cannot be explained. Model output explanation has been proposed as an approach for explainable artificial intelligence^33^, to explain the rationale for predicting the output of deep learning. Grad-CAM and guided Grad-CAM are class activation mapping methods that use gradients and are often used in deep learning of medical images^34,35^. In the Grad-CAM used in this study, the focus area was the contact between the inferior alveolar canal and the mandibular third molar or the closest site in both the contact and continuity analyses. In other words, it is likely that learning is possible with an understanding of the exact feature area. However, it is difficult to understand the characteristic areas at more detailed points, such as the defect of the cortical bone on the upper wall of the inferior alveolar canal. Thus, further research is required to examine the approach of explainable AI with guided backprop^36^.

This study has several strengths. First, we analyzed the positional relationship between the inferior alveolar canal and the mandibular third molar using panoramic radiographs. We evaluated the continuity of the inferior alveolar canal with contact and CT findings as correct labels from the panoramic radiograph findings. This study was the first to use the same image to determine the ability to classify positional relationships using panoramic radiographs as screening and determine whether to classify the position of the inferior alveolar nerve in three dimensions as a potential evaluation of deep learning. Second, this study is the first to introduce effect size as a statistical evaluation method for the performance metrics of each CNN model that analyzes the positional relationship between the inferior alveolar canal and mandibular third molar. The effect size indicates effectiveness of an analytical operation and the strength of the association between each variable^37^. Therefore, the detected effect size is an essential prior parameter to help estimate the sample size in studying the relationship between the inferior alveolar canal and mandibular third molar using deep learning.

This study had two limitations. The first is that the data were collected from a single facility and were not validated externally. Internal validity was evaluated using confidence intervals from the dataset via mutual validations. However, to satisfy the external validity criterion, more data must be used in multicenter joint research. The second is the use of only two CNN models. In this study, we analyzed the data using ResNet50 and ResNet50v2. By examining previously published CNN and original CNN models, it may be possible to identify a better model for classifying the relationship between the inferior alveolar canal and the mandibular third molar.

## Conclusions

In this study, we investigated the effects of a deep learning model using contact and continuity analyses to classify the positional relationship of the inferior alveolar canal. Contact analysis classified the presence or absence of contact with the mandibular third molar on panoramic radiographs, and continuity analysis classified the presence or absence of bone continuity in the inferior alveolar canal on CT images. CNN models showed a high performance in contact analysis. The CNN deep learning model using SAM as the optimizer for ResNet50v2 exhibited the highest performance. However, the continuity analysis did not show high classification performance. These results indicate that deep learning plays a vital role in primary screening using panoramic radiographs to evaluate the positional relationship between the inferior alveolar canal and the mandibular third molar. However, further studies are needed on deep learning to replace CT imaging for 3D evaluation.

## Supplementary Information

Supplementary Information.

## Data Availability

The image data are not publicly available due to privacy. The other statistical data in this study are available on request from the corresponding author.
