# Supplementary material for: Deep learning model for analyzing the relationship between mandibular third molar and inferior alveolar nerve in panoramic radiography
Source: Sci Rep. 2022 Oct 8;12:16925. doi: 10.1038/s41598-022-21408-9 (PMC9547920; doi:10.1038/s41598-022-21408-9)
Supplement: Supplementary file 1 — Supplementary Information. [file 41598_2022_21408_MOESM1_ESM.docx]

Deep learning model for analyzing the relationship between mandibular third molar and inferior alveolar nerve in panoramic radiography

Shintaro Sukegawa ^1,2,^ *, Futa Tanaka ^3^, Takeshi Hara ^3, 4^, Kazumasa Yoshii ^3^, Katsusuke Yamashita ^5^, Keisuke Nakano ^2^, Kiyofumi Takabatake ^2^, Hotaka Kawai ^2^, Hitoshi Nagatsuka ^2^, Yoshihiko Furuki ^1^

^1^ Department of Oral and Maxillofacial Surgery, Kagawa Prefectural Central Hospital, 1-2-1, Asahi-machi, Takamatsu, Kagawa 760-8557, Japan

^2^ Department of Oral Pathology and Medicine, Graduate School of Medicine, Dentistry and Pharmaceutical Sciences, Okayama University, Okayama 700-8558, Japan

^3^ Department of Electrical, Electronic and Computer Engineering, Faculty of Engineering, Gifu University, 1-1 Yanagido, Gifu, Gifu 501-1193, Japan

^4^ Center for Healthcare Information Technology (C-HiT), Tokai National Higher Education and Research System, 1-1 Yanagido, Gifu, Gifu 501-1193, Japan

^5^ Polytechnic Center Kagawa, 2-4-3, Hananomiya-cho, Takamatsu 761-8063, Kagawa

**Appendix**

***Learning rate scheduler***

In this study, a learning rate scheduler was used for efficient deep learning.

**
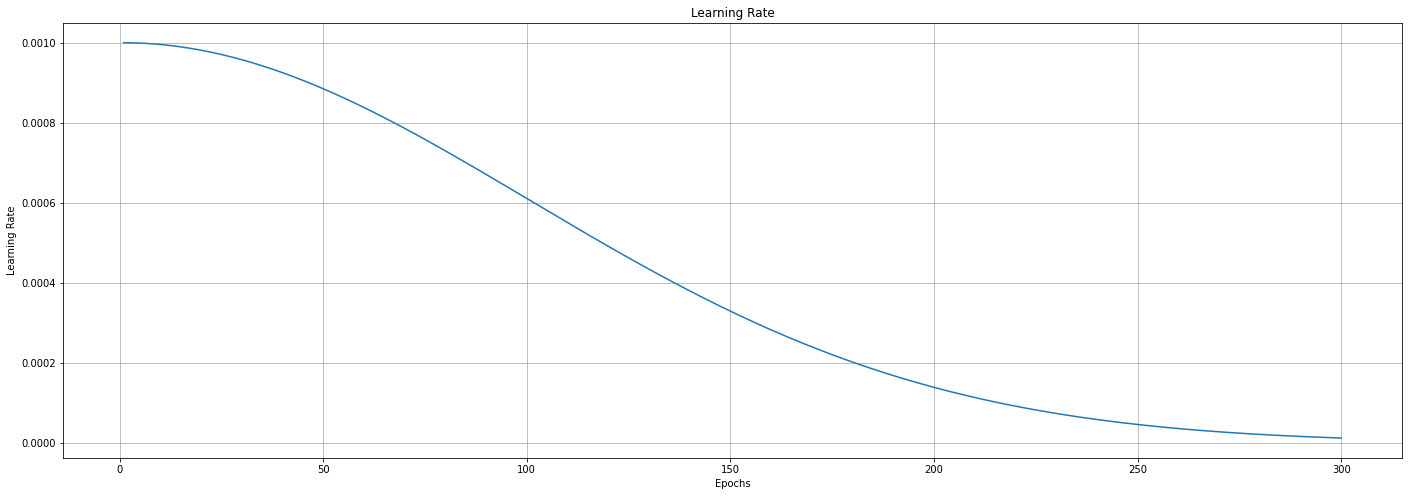
Fig. Change in learning rate for each number of epochs in scheduler time based decay as a learning rate in this study**

***Performance metrics***

We evaluated the performance metrics, with accuracy, precision, recall and the F1 score as defined in Equations 3–7, along with the receiver operating characteristic curve (ROC), and the area under the ROC curve (AUC).

Accuracy is the ratio of correct predictions. Precision is the ratio of true positives to true positives and false positives. Recall is the ratio of true positives to true positives and false negatives. F1 score is a harmonic mean of precision and recall.

The ROC curves were shown for the complete dataset from the 10-fold cross-validation, producing the median AUC value.

ROC curve shows the true-positive rate, or sensitivity, against its false-positive rate, or 1 − specificity, as a function of varying discrimination thresholds.

In followed equations, TP, TN, FP, and FN represent true positive (normal correctly identified), true negative (abnormal correctly identified), false positive (abnormal incorrectly identified), and false negative (normal incorrectly identified) results, respectively.

$accuracy=\frac{TP + TN}{TP + FP + TN + FN}$ (1)

$precision=\frac{\mathrm{TP}}{TP + FP}$ (2)

$recall=\frac{\mathrm{TP}}{TP + FN}$ (3)

$F1 score=2\times\frac{precision \times recall}{precision + recall}$ (4)
